# Formative experience and stock price crash risk: Evidence from China’s “Down to the Countryside” movement

**DOI:** 10.3389/fpsyg.2022.971101

**Published:** 2022-10-14

**Authors:** Maoguo Wu, Yuang Zhang, Dongkun Shi

**Affiliations:** ^1^SILC Business School, Shanghai University, Shanghai, China; ^2^China CITIC Bank, Changzhou, China

**Keywords:** early life experience, Down to the Countryside movement, educated youth, stock price crash risk, corporate risk-taking

## Abstract

This study explores the influence of chairpersons’ early life experiences during the “Down to the Countryside” movement—a unique social phenomenon in China—on company stock price crash risk. This study uses 2007–2018 data from China’s A-share listed companies and a multiple regression analysis model to assess the influence of chairpersons’ Down to the Countryside experiences on the stock price crash risk of their corresponding companies. The empirical results demonstrate that chairpersons’ “sent-down” experiences shaped their risk-aversion management style, reducing companies’ risk-taking capacity. Consequently, this formative experience reduces stock price crash risks and increases company value. Heterogeneity tests on companies with different property rights reveal that chairpersons’ sent-down experiences are more pronounced in non-state-owned companies, as against state-owned companies. To alleviate endogeneity issues (e.g., self-selection bias and omitted variables) various endogeneity controls (e.g., propensity score matching, placebo tests, and difference-in-differences (DID) tests) were conducted. The results also persisted in a series of robustness checks. This study provides a new perspective on the relationship between chairpersons’ early life experiences and stock price crash risks. The empirical evidence has implications for the recruitment, incentivization, and supervision of chairpersons.

## Introduction

Over 40 years after its economic reform and the implementation of its opening-up policy, China has become the backbone of global economic development and its worldwide political status has been gradually rising. During this period, outstanding entrepreneurs have emerged and contributed indelibly to China’s national economic development, witnessing and playing a part in China’s economic miracle. Among these entrepreneurs, a unique subgroup has evolved with the distinctive characteristics of being firm, tenacious, prudent, cautious, and possessing a sense of gratitude (Fan, 2005). They overcame the difficulties of the early stages of the economic reform and worked together to construct a pathway for the rapid and stable development of China’s national economy (Liang and Li, 2013). Moreover, these entrepreneurs share the experience of the “Up to the Mountains and Down to the Countryside” movement and are consequently referred to as *Zhiqing* (“the educated youth” or “sent-down youth”).

Extant literature has extensively investigated the impact of the personal characteristics of top managers on corporate decisions. Apart from age (Huang et al., 2012; Yim, 2013; Jenter and Lewellen, 2015), gender (Gul et al., 2011; Srinidhi et al., 2011), educational background (Bertrand and Schoar, 2003), and miscellaneous personal traits (Wu and Wu, 2008; Jiang et al., 2009; Li et al., 2011; Zhang et al., 2011), early life experience that has an important and lasting influence on a person’s character and attitudes, the formative experience, also influences top managers’ corporate strategies and decision making. Previous research has investigated executives’ personal experiences such as military experience (Benmelech and Frydman, 2015; Law and Mills, 2017), financial experience, poverty experience (level of poverty at birth or childhood experience of the “Great Famine”) (Shen and Xing, 2014; Zhao and Yan, 2015; Wang and Cao, 2017; Cui et al., 2022), experience of the Great Depression or economic downturn (Malmendier et al., 2011; Schoar and Zuo, 2017), early-life exposure to fatal disasters (Bernile et al., 2017), and possession of pilot licenses (Cain and McKeon, 2016). Few studies have focused on the influence of executives’ experience of the Down to the Countryside movement, a unique political event in China, on business management behavior. Kendler et al. (2002) examined the relationship between individuals’ early experiences and neuroticism, and found that early life experiences, particularly catastrophic failures, had long-term effects on individuals’ pre-dispositional diathesis. Shi (2010) documented that the impact of early life experiences changed individuals’ propensity for risk and their underlying cognition and belief about the future. As such, the “Down to the Countryside” movement—a distinctive social phenomenon—shaped the unique characteristics of the “educated youths.” The Down to the Countryside movement originated in the former Soviet Union. In its large-scale land reclamation campaign in 1954, it changed the previous approach of moving people to other places for land reclamation. Instead, it made urban youth the main body of land reclamation. After the collapse of the former Soviet Union, Down to the Countryside became a unique social phenomenon in China. Therefore, the individual characteristics of top managers shaped by Down to the Countryside experiences are unique to China. Existing studies have mainly explored individuals’ Down to the Countryside experiences from sociological and historical perspectives, analyzing the effect on perceived well-being (Liang, 2015), social trust (Liang and Li, 2013), income (Wang, 2011), and education (Ye, 2011). However, few studies have investigated the economic impact of the experiences or this unique social phenomenon in the corporate setting.

Stock price crash risk is a potential economic risk that measures the probability of a company’s stock price plummeting in the future. Companies with higher stock price crash risk are more likely to experience a stock price slump. There have been a series of incidents of “flash crashes” in the history of China’s capital market. For instance, from June to July 2015, the Shanghai Stock Exchange Composite Index (SSE Index) plummeted for 17 consecutive trading days. In August of the same year, following a short period of stability, the SSE Index experienced another consecutive drop of 1,000 points, setting a 20-year plunge speed record for China’s capital market. In 2020, owing to the economic downturn caused by the COVID-19 pandemic, China maintained prolonged quantitative easing measures. With the continuous recovery of China’s economy, various monetary policies have gradually become normalized, and further liquidity tightening is likely to trigger turbulence in the capital market. Thus, stock price crash risks have a vast impact on companies and investors as well as on the stability of the capital market. Therefore, it is of utmost importance to uncover the factors affecting stock price crash risk, identify individual and social factors, and establish measures to reduce risk.

During the Down to the Countryside movement, the educated youths transformed from economically well-off students to subsistence farmers, and they started their first jobs with a sense of reluctance, which may make them behave cautiously and with low risk tolerance in their future work (Bonnin, 2005; Liang, 2015). The educated youths, who had lived in cities with relatively superior conditions, but experienced firsthand the hardships of living in the countryside during their time, are more grateful and contented in the face of the hard-won national and personal development achievements over the years (Interview Record Office of the Central Party School, 2017). They have a stronger sense of mission, higher moral standards, and a stronger sense of social responsibility than other generations (Fan, 2005). Chairpersons with Down to the Countryside experiences went through negative shocks such as interruption of education, tough living environment, and hard work. The experiences made educated youths conditioned to fear the complex macro environment and averse to the uncertainty of expectations. Therefore, they also behave more cautiously in corporate decision making. Although the forcible Down to the Countryside experience has brought the educated youths a sense of reluctance and reduced their happiness and risk tolerance, it has also shaped precious qualities such as contentment and gratitude, self-improvement, and pragmatism, which were reflected in their work decisions when they took up their jobs. For the educated youths who grew up to be the chairpersons of their corresponding companies, do the personal traits and risk appetite shaped by early life experiences affect corporate decision making? From the perspective of stock price crash risk, this study explores the underlying mechanism of the impact of chairpersons’ Down to the Countryside experiences on their corporate decision-making behavior.

This study used 2007–2018 data from China’s A-share listed companies and a multiple regression analysis model to assess the influence of chairpersons’ Down to the Countryside experiences on the stock price crash risk of their corresponding companies. Empirical results demonstrate that the “sent-down” experience shaped chairpersons’ risk-aversion management style, reducing companies’ risk-taking capacity. Consequently, this formative experience reduces stock price crash risk and increases company value. Heterogeneity tests on companies with different property rights reveal that chairpersons’ sent-down experiences are more pronounced in non-state-owned companies, as against state-owned companies. To alleviate endogeneity issues such as self-selection bias and omitted variables, a variety of endogeneity controls, such as propensity score matching, placebo tests, and difference-in-differences (DID) tests were conducted. The results also persist in a series of robustness checks. This study may help extract and explore the unique elements behind the financial behavior of Chinese enterprises, help investors deepen their understanding of the quality of financial information of listed companies from the perspective of chairpersons’ early life experiences, and improve the efficiency of resource allocation in China’s capital market. The findings have key theoretical and practical implications concerning chairpersons’ recruitment, incentivization, and supervision. Specifically, this study provides a new perspective on the relationship between chairpersons’ early life experiences and stock price crash risk.

The remainder of this paper is organized as follows: Section “Related literature” reviews related literature. Section “Hypotheses” develops the hypotheses. Section “Research design” is devoted to the research design with an introduction of the data and variables. Section “Results” presents the empirical results. Section “Conclusion” concludes the paper.

## Related literature

### Effect of the Down to the Countryside movement on individuals

The Down to the Countryside movement was a significant political event in the history of China, and embedded the zeitgeist within people who experienced it. This policy was launched in 1968 in response to Chairman Mao’s instructions in the People’s Daily, the party’s leading newspaper, which stated that “educated youths must go to the countryside and receive re-education from the poor, lower-, and middle-class peasants” (People’s Daily, 1968). During the implementation of the policy, which lasted for over a decade, approximately 17 million urban junior and senior high school and college students left the city for the countryside. These students were resettled in impoverished rural areas and spent their youth in the so-called “broad world” of the countryside. As the first generation after the founding of the People’s Republic of China, they shouldered the extraordinary mission of the times and experienced great changes in their lives. These experiences left profound psychological and behavioral characteristics that were distinctive of that time (Ye, 2011).

This large-scale movement had a unique social background. Specifically, the movement occurred in the early stage of the Cultural Revolution, when the social environment had not yet stabilized. The rise in unemployment caused by the Cultural Revolution made it impossible for recent graduates to find employment through regular channels. Thus, newly graduated students were fearfully propelled into the uncertainty of a complex social environment. When the Down to the Countryside movement began, these youths, driven by the ideal of maturing and progressing in the countryside, voluntarily left their economically developed cities and moved to economically undeveloped rural areas. However, the harsh living conditions and low productivity in rural areas were worse than anticipated. The vast majority of them were engaged in routine manual labor in the fields and on the farms on a daily basis. Such a reality negatively affected the will of the youths who had left their families and schools behind, generating a plethora of negative emotions, such as loneliness and confusion (Bonnin, 2005; Zheng, 2013; Liang, 2015). As adolescence is a critical developmental stage in advancement to adulthood, in which values and perspectives are formed, negative experiences during this period may lead to severe psychological trauma and a sense of inferiority (Main et al., 1985). Additionally, the psychological impact of adolescent experiences may have a prolonged influence on future life and work. Wang (2011) found that the perceived well-being of educated youths, particularly those who experienced the early stage of the movement, exhibited lower happiness than people who did not have such experiences.

As education in urban areas was interrupted during the movement, the expected life plans of the youths were also disrupted, and expectations of continuing education were essentially eradicated. Although these youths went to rural areas of their own accord, they lacked a viable alternative after witnessing the contradictions of the various Red Guard units^1^ and their negative impacts on society. When the educated urban youths first arrived in rural areas, owing to differences in educational background and lifestyle, they experienced several challenges when interacting with the rural farmers. Additionally, the limited quota for returning to the cities triggered malicious competition among the educated youths, resulting in societal distrust toward both the government and each other (Liang and Li, 2013).

Despite the negative emotions, positive factors emerged that shaped the characteristics of these youths. The resettlement from well-developed cities to undeveloped rural areas transformed individuals from pampered to progressive, generating an understanding of the difficulties of rural life experienced by farmers. Hence, the educated youths who experienced the relocation were more likely to feel gratitude toward and satisfaction with their urban environment, compared with their non-relocated counterparts (Interview Record Office of the Central Party School, 2017). Additionally, many of the educated youths undertook non-agricultural tasks, such as becoming agricultural technicians, “barefoot doctors,^2^” and teachers. Thus, they served as a bridge between the rural and urban areas and contributed to the development of agriculture and rural education. As such, the resettlement experience played a powerful role in shaping their values and helping them develop a strong sense of mission, social responsibility, and high moral standards (Fan, 2005).

### Stock price crash risk

Following the 2008 financial crisis, a great deal of research pertains to the potential determinant factors of stock price crash risk. Current studies mainly focus on the quality of accounting information, company and management characteristics, and investment participants.

The concept of stock price crash risk was first proposed by Romer (1993), who argued that stock price crashes originated from often-concealed bad news during the early life of companies; once the news was finally disclosed, the stock price experienced a considerable decline. Financial reports serve as a reference for investors and are expected to represent the actual financial situation of a company. Therefore, their accurate disclosure has a significant influence on stock price crash risk. Hutton et al. (2009) found that companies with less financial transparency are more likely to conceal bad news, which increases the risk of a stock price crash, whereas greater information transparency effectively reduces the ability to conceal bad news and the risk of a stock price crash. Jiang (2015) and Ye et al. (2015), using data from Chinese listed companies, investigated stock price crash risk from the perspective of accounting information comparability and internal control of information disclosure. They both found that better information disclosure leads to lower stock price crash risk.

Additionally, the principal-agent problem of management affects information disclosure. Jin and Myers (2006) argued that managers acting exclusively in their own interest are more likely to attempt to conceal bad news compared with managers acting more stringently. However, when the scale of the bad news exceeds what management can effectively suppress, the stock price tends to plummet.

Some studies have focused on capital market participants, concentrating on the impact of institutions’ and analysts’ forecasts and the influence of media attention on the risk of stock price crashes. As important participants in the capital market, institutional investors play an important role in supervising enterprises, which hinders information asymmetry, thereby reducing the risk of stock price crashes. Furthermore, the “herd effect” (Xu et al., 2013) and analysts’ optimism (Xu et al., 2012) lead to greater stock price crash risk and are more pronounced when the proportion of institutional to private investment is high.

Moreover, top managers’ personal characteristics may affect stock price crash risk. Li and Liu (2012) explored the relationship between the CEOs’ and CFOs’ gender and stock price crash risk and found that companies with female CEOs tend to have significantly lower stock price crash risk, compared to those with male CEOs. This effect is more prominent among companies with older female CEOs who possess greater centralized authority, compared with companies with younger CEOs. However, given that the CFO is subject to the power of the CEO, the gender of the CFO does not appear to have a significant effect. Other studies have found that the self-confidence of executives may also impact the risk of future stock price crashes (Kim et al., 2015). Overconfident (vs. underconfident) managers are more likely to believe that they are able to resolve bad news and more inclined to make aggressive decisions to conceal it, which leads to greater stock price crash risk when the concealment fails. Cao et al. (2019) found that board directors with foreign experience (BDFEs) help reduce stock price crash risk. The negative association between BDFEs and crash risk is more pronounced for firms with more agency problems, weaker corporate governance, and less overall transparency. Chen et al. (2021) found that firms led by CEOs with early-life disaster experience have higher stock price crash risks. The findings are consistent with CEOs who experienced early-life disasters being more risk tolerant, and thus more willing to accept the risks associated with bad news hoarding, engendering the formation of stock price crashes. Cheng et al. (2021) found that CEOs who experienced the Great Famine during early life tend to hoard bad news, which results in higher stock price crash risks. The more severe and prolonged the Great Famine that the CEOs experienced, the greater the effect of this traumatic experience. CEOs’ decision-making power enhances the adverse effect of their early-life traumatic experiences on crash risks. Cui et al. (2022) found that top executives’ famine experience significantly reduces firms’ stock price crash risk. Firm characteristics and external monitoring mechanisms affect the relationship. In addition, these firms have higher information transparency and lower information risk, which ultimately reduce future stock price crash risks.

## Hypotheses

The upper echelon theory (Hambrick and Mason, 1984) states that individuals in power are the key decision-makers and strategy-planners in their organizations, and have a significant impact on their companies’ development and potential risks. Managers’ decision-making processes are impacted by their cognitive foundations. Managers whose vision was limited in early experiences have limited adoption of strategies among perceived alternatives. Additionally, managers’ personal values have a direct impact on corporate strategy choices. Thus, this establishes a chain of evidence that reflects the influence of managers’ experiences during adolescence on their attitudes, values, and personalities, which affect future behavior. This line of effects forms information filters when making corporate decisions and greatly affects the decisions made. Zhao and Yan (2015) argued that demographic characteristics (like age and tenure) alone cannot explain differences in managers’ decision-making. Wang and Cao (2017) argued that to uncover the defining features of corporate decision-making, executives’ formative experiences, particularly the social influences of their early lives, require examination.

The Down to the Countryside movement was initiated by political decree and began during the Cultural Revolution. The macro-environment at the time was characterized by large changes, as factories were closed down, workers became unemployed, and students discontinued their education. Thus, the countryside became the first workplace for many educated youths, and the organizational forms and atmosphere in the countryside reflected the nature of the organizational environment. Given the differences in the environment and living habits between urban and rural areas, the relocated youths were forced to continuously adapt their attitudes and living styles to their micro-environment. These macro-, organizational-, and micro-environmental factors had a consistent effect on the youths’ development as they were faced with continuous changes in their surroundings. As such, being part of the Down to the Countryside movement became an archetypal experience during the early development of that generation.

Individuals included in the Down to the Countryside movement were mostly aged 16 to 19 years and were students or recent graduates from junior and senior high schools and colleges; as such, they were adolescents or in early adulthood. According to the personality development theory, this age is a sensitive period in forming identity and the ability to be intimate with others. However, the social environment during the Cultural Revolution, with factories and schools closing down, caused significant confusion and a loss of orientation in life, seriously affecting educated youths’ identity. Fan (2005) argued that the Down to the Countryside experience was both a physical and mental challenge for educated youths, resulting in emotional and cognitive changes. Li and Zhu (2009) revealed that the experience had long-term effects on individuals’ behavioral and thinking styles in adulthood. This experience of encountering social change and having to leave behind the prospects of further education and plans during this age of rapid development may have increased the educated youths’ likelihood of developing an aversion to changes in the macro-environment and avoidance of surrendering control of their future as they age. Schoar and Zuo (2017) argued that the macroeconomic environment of when individuals first enter the labor market has a key impact on their propensity to risk in decision-making. Individuals who have experienced a lethargic economic environment are more cautious and conservative toward work-related risks. Given the chaotic environment during the Down to the Countryside movement, the economy and labor market were severely impacted. As such, educated youths first entered the rural labor market and experienced confusion regarding the best way to manage the underdeveloped environment and social conditions. Such experiences may have affected their later propensity to risk aversion at work.

Tremendous physical and mental stress was experienced by the relocated youths owing to their interrupted studies, dissolution of their academic goals, and being separated from parents and schoolmates to work in harsh rural conditions and earn “work points^3^” through hard physical labor each day. Such stress may create a “victim mentality” (Main et al., 1985), which may dampen the determination to excel in life and cause negative emotions, such as a sense of loneliness and confusion or a reduced sense of well-being and risk tolerance (Shi, 2010). The experience may have instilled a sense of loneliness and uncertainty. As these youths were in an important period in the formation of their personality, particularly in their sense of identity and intimacy with others, the experience may have left a negative effect on their confidence and personality. According to the personality development and imprinting theory, the personality, cognition, and values shaped during adolescence tend to remain throughout a person’s life, affecting work and management in their later careers (Bernile et al., 2017). The rapid change of identity may bring fear of the unknown and aversion to uncertainty, creating additional cautiousness in future work. Thus, when faced with the decision to either disclose information or conceal it, chairpersons with Down to the Countryside experience (vs. those without) are expected to be more likely to be pessimistic about the consequences and develop an inclination toward disclosure to avoid the uncertainty of concealment.

Despite the negative shocks for the educated youths, the positive influences of the Down to the Countryside experiences may have shaped top managers’ characters and decision-making. Previous studies have revealed that the experience has led to increased gratitude and contentment (Interview Record Office of the Central Party School, 2017), as well as a stronger sense of mission, social responsibility, and moral standards (Fan, 2005). As such, executives with these experiences (vs. those without) are expected to be less likely to be influenced by the principal-agent effect. Chairpersons are defined here as individuals entrusted by the board of directors to serve as the primary person in power of an enterprise; therefore, it is expected that chairpersons with Down to the Countryside experiences (vs. those without) are more grateful for such trust and have a stronger sense of responsibility toward their shareholders and higher moral standards. Hence, in the face of bad news that negatively impacts the company, such chairpersons are more inclined to disclose information, increasing information transparency for the board of directors and shareholders, and reducing the risk of stock price crashes caused by information concealment. Therefore, the following hypothesis (H) was formulated:

*H1: Other factors remaining constant, chairpersons’ Down to the Countryside experiences reduce stock price crash risk.*

From 1978 onward, most of the educated youths returned to their original cities, and only a small proportion remained in the countryside. However, many of those who returned struggled to adapt to city life. Specifically, as the cities had undergone substantial changes during their absence, it was difficult for these technically unskilled workers to compete in the labor market with the incompatible farming skills they had acquired. There were limited positions available in state-owned enterprises (SOEs) which they could fill, and most were too old to join the army. Therefore, many were forced into positions of basic labor in sub-district factories or neighborhood production teams. Those who succeeded in gaining a position in the SOEs were considered lucky and tended to value their hard-won opportunity (Chen and Zha, 2005). However, it is suspected that luck was not the key element that landed them their positions; rather, their indomitable determination formed during their Down to the Countryside experiences played a more significant role. Having experienced the frustration of being sent away from home, it is expected that the returned youths were more likely to value development opportunities and be more grateful for their achievements, shaping a more effective working style.

Following the economic reform and to achieve economic transformation and other political goals, all government levels were involved in the selection, motivation, and supervision of SOE managers through administrative intervention (He and Zhang, 2015). Therefore, a certain proportion of managers in SOEs are former government workers, considered “official” managers. These managers are driven more by political promotions and prefer to act cautiously during their tenure to ensure “an uneventful start and end” of their position (Jiang et al., 2009). Non-SOEs, as market-oriented organizations, select their managers by following the rules of the labor market and adopt measures to motivate managers, such as equity incentives and annual assessments. However, as outlined in the principal-agent problem, to obtain medium- and long-term benefits, such managers may leverage a high degree of information asymmetry in market-oriented companies to gain shareholder trust by creating an illusion of sound operations. This is typically done by concealing bad news and revealing good news that is not yet finalized to increase compensation and market reputation (Kothari et al., 2009; Zhang et al., 2011). Thus, it is expected that the different forms of business ownership affect management style. Hence, the following hypothesis was proposed:

*H2: A negative correlation between chairpersons’ Down to the Countryside experiences and stock price crash risk is more pronounced in state-owned enterprises than in non-SOEs.*

Comparatively speaking, with the increase in age, chairpersons’ understanding of and ability to adapt to changes tends to decline, impacting their ability to analyze and invest in new projects. Particularly, as retirement approaches, executives tend to prefer to maintain the status quo. Wu and Wu (2008) found that younger executives are more sensitive to new investment opportunities and more willing to formulate strategic plans, such as research and development (R&D) and innovation, resulting in greater operational risks. However, executives with a longer (vs. shorter) tenure may be more aware of the areas of production and operations that require improvement and are more willing to adopt an open attitude for innovation and work improvement. Hambrick et al. (1996) asserted that executives’ tenure has an impact on the adoption of more aggressive innovation strategies by the company. Liu and Liu (2007) found that executives with a longer (vs. shorter) tenure are more inclined to increase R&D expenditure. They concluded that tenure and R&D expenditure are positively correlated. Whether chairpersons are approaching retirement or facing reappointment makes a difference in their current business decisions and affects stock price crash risks. Furthermore, as discussed previously, the risk of stock price crashes often originates from the concealment of bad news. Sound internal governance ensures information disclosure in a timely manner, thereby allowing both good and bad news to receive equal weight when announced. As chairpersons with Down to the Countryside experiences tend to have a higher sense of mission and responsibility, these qualities are better exerted in well-governed enterprises, which reduces stock price crash risks. As such, the following hypotheses were proposed:

*H3: A negative correlation between chairpersons’ Down to the Countryside experiences and stock price crash risk is more pronounced among chairpersons not approaching retirement than among those approaching retirement.*

*H4: A negative correlation between chairpersons’ Down to the Countryside experiences and stock price crash risk is more pronounced among chairpersons not likely to be re-appointed than among those facing reappointment.*

*H5: A negative correlation between chairpersons’ Down to the Countryside experiences and stock price crash risk is more pronounced in companies with poor, rather than good, governance practices.*

## Materials and methods

### Research sample and data source

Main board-listed companies in the SSE and the Shenzhen Stock Exchange (SZSE) between 2007 and 2018 were selected for the empirical analysis. Financial data of the sample companies were extracted from the China Stock Market and Accounting Research (CSMAR) database. A comprehensive data mining method was adopted to cross-compare chairpersons’ information between the CSMAR, the Wind Economic Database, and Baidu Baike^4^ to supplement any information missing from the initial search and obtain a more comprehensive dataset. The collected data were processed according to the following principles:

(1) Financial institutions, insurance companies, and companies with special treatment status (ST and *ST) were excluded from the dataset.
(2) As reported annual earnings during the initial public offering (IPO) period tend to differ greatly from that during regular operating periods, the IPO year was excluded for all companies in the dataset.
(3) To alleviate the potential influence of outliers on the conclusion, all continuous variables in the dataset were winsorized at the 1st and 99th percentiles.

### Variable definitions and research model

#### Stock price crash risks

Based on the method proposed by Kim et al. (2011), stock price crash risk (*CRASHRISK*) was calculated as outlined below. First, using Equation (1), the specific return of stock *i* in week *t*, denoted as *W*_*i,t*_ and ε_*i*,*t*_, was defined as the residual term of Equation (2).


(1) $$W_{i,t}=l\,n\,\left(1+\varepsilon_{i,t}\right)$$

(2) $$r_{i,t}=\alpha_{i}+\beta_{1,i}\times r_{m,t-2}+\beta_{2,i}\times r_{m,t-1}+\beta_{3,i}\times r_{m,t}$$

$$+\beta_{4,i}\times r_{m,t+1}+\beta_{5,i}\times r_{m,t+2}+\varepsilon_{i,t},$$

where *r*_*i,t*_ is the return of *i* in *t* and *r*_*m,t*_ is the return of the weighted average market capitalization in *t*. To reduce the return deviation caused by asynchronous trading, the lag and lead terms of *r*_*m,t*_ were added in Equation (2).

Next, the negative return skewness coefficient (*NCSKEW*) and up and down volatility (*DUVOL*) were calculated to measure *CRASHRISK*, as shown in Equations (3) and (4), respectively. A greater value indicates a greater stock price crash risk:


(3) $$NCSKEW_{i,t}=-\left[n{\left(n-1\right)}^{3/2}\sum W_{i,t}^{3}\right]/\left[\left(n-1\right)\,\left(n-2\right)\,{\left(\sum W_{i,t}^{2}\right)}^{3/2}\right],$$

where *n* is the number of trading weeks per year for *i*.

The calculation of *DUVOL* is shown in Equation (4):


(4) $$D\,U\,V\,O\,L_{i,t}=l\,o\,g\,\left\{\left[\left(n_{u}-1\right)\,\sum_{D\,O\,W\,N}W_{i,t}^{2}\right]/\left[\left(n_{d}-1\right)\,\sum_{U\,P}W_{i,t}^{2}\right]\right\}.$$

Based on whether the rate of return (RoR) *W*_*i,t*_ of a given week was greater than the average weekly RoR of the year, the weeks in a given year were divided into “DOWN WEEK” (<average weekly RoR) and “UP WEEK” (>average weekly RoR). The values of UP WEEK and DOWN WEEK are denoted as *n_u_* and *n_d_*, respectively.

#### Explanatory variables

Previous research on the impact of specific macro-events, such as the Great Depression (Malmendier et al., 2011) and the Chinese Great Famine (Shen and Xing, 2014), on people has frequently adopted birth year to infer whether an individual was involved in the event. The participants of the Down to the Countryside movement were students graduating from urban junior and senior high schools, and schools accepted students based on their age by September of that school year. As such, the year of birth is insufficient, as it does not denote which school year an individual was in. In December 1968, the government implemented the mandatory Down to the Countryside policy, and implementation was not relaxed until the restoration of the college entrance examination system in 1977. Particularly, the movement continued between 1968 and 1977, when more than 98% of the educated youths were sent to the countryside. Considering that the policy originally intended to cover junior and senior high school students who graduated in 1966, 1967, and 1968, as its implementation began in 1968, we expanded the period from 1966 to 1977. According to the school admission age from 1950 to 1980 (starting from seven years old; Gong et al., 2015), it was inferred that the oldest students who graduated in 1966 were 19 years old, and the youngest students who graduated in 1977 were 16 years old; their corresponding birth years are between 1949 and 1960. Following Malmendier et al. (2011) and Shen and Xing (2014), a dummy variable (*Zhiqing*) was introduced to indicate whether a chairperson was involved in the movement. If the chairperson’s birth date was between September 1948 and August 1960, *Zhiqing* took the value of 1; otherwise, it took the value of 0. Individuals with rural household registrations^5^ or with foreign, Hong Kong, or Macau identification documents were categorized as non-*Zhiqing* and the variable *Zhiqing* was assigned 0 judging from their birthplaces and education background. Companies that underwent changes in chairpersons in a given year were identified. *Zhiqing* was assigned 1 if the chairperson had Down to Countryside experience and was in the position for more than half of a year. The definitions of other control variables are presented in Table 1.

**TABLE 1:** Variables and definitions.

Variable type	Variable	Definition
Explained variable	*NCSKEW*_*t*_	Negative return skewness coefficient (*NCSKEW*) in year *t* [see Equation (3) for the calculation].
	*DUVOL*_*t*_	Up and down volatility (*DUVOL*) in year *t* [see Equation (4) for the calculation].
Core explanatory variable	*Zhiqing*	1 = Birth date is between September 1948 and August 1960; 0 = otherwise.
Personal characteristics	*Gender*	1 = male; 0 = female.
	*Edu*	Technical secondary school or lower level = 1, junior college = 2, bachelor = 3, master = 4, Ph.D. = 5, others (such as honorary doctorate and correspondence degree) = 6, and MBA/EMBA = 7.
	*Age*	Age of the chairperson.
	*TENURE*	The natural logarithm of the chairperson’s tenure in months.
	*INSIDERATE*_*t–1*_	The proportion of inside directors in year (*t-1*).
	*DUALt-1*	The chairperson and general manager are the same person = 1; otherwise = 0.
Company features	*SOE*_*t–1*_	The company is state-owned in year (*t-1*) = 1; otherwise = 0.
	*DTURN*_*t–1*_	The monthly average excess turnover rate of stock *I* in year (*t-1*) is the difference between the monthly average turnover rates in year *t* and (*t-1*).
	*SIGMA*_*t–1*_	The return volatility of stock *I* in year (*t-1*) is the standard deviation of weekly specific return in the same year.
	*SIZE*_*t–1*_	Natural logarithm of total assets at the end of year (*t-1*).
	*MB*_*t–1*_	Market-to-book ratio in year (*t-1*).
	*LEV*_*t–1*_	Asset-liability ratio in year (*t-1*).
	*ROA*_*t–1*_	Earnings before interest and taxes for year (*t-1*) / total assets at the end of the period.
	*ABACC*_*t–1*_	Absolute value of discretional accruals in year (*t-1*) calculated based on the modified Jones model.
	*PPE*_*t–1*_	Net fixed assets in year (*t-1*) / total assets at the end of the period.
	*RET*_*t–1*_	Average weekly specific return in year (*t-1*).
Fixed Effect variable	*Firm*	Dummy variable for firm.
	*Year*	Dummy variable for year.
	*Industry × Year*	The interaction term of the dummy variable for industry and dummy variable for year.

### Empirical model

To test the hypotheses, this study constructed an ordinary least squares multiple regression model for the empirical analysis, as follows:


(5) $$C\,R\,A\,S\,H\,R\,I\,S\,K_{i,t}=\beta_{0}+\beta_{1}\times Z\,h\,i\,q\,i\,n\,g_{i,t-1}$$

$$+\beta_{i}\times C\,o\,n\,t\,r\,o\,l\,s_{i,t-1}+F\,i\,x\,e\,d\,e\,f\,f\,e\,c\,t\,s+\varepsilon_{i,t}.$$

*CRASHRISK* was defined as the dependent variable, which includes two calculation methods (*NCSKEW* and *DUVOL*), and *Zhiqing* was the core explanatory variable. *Controls* denoted a vector of control variables listed above, while *Fixedeffects* symbolized the fixed effect control variables, such as the fixed effects of the firm, year, and industry for each year. ε was defined as the residual. Additionally, standard errors were clustered at the firm level and robust standard error adjustment was performed.

## Results

### Descriptive statistics

Table 2 reports the distribution of the core explanatory variable (*Zhiqing*) by year. Prior to 2012, *Zhiqing* accounted for more than 50% of the total observations for each year, while the proportion of non-*Zhiqing* became larger following 2012. A total of 10,213 chairpersons experienced the Down to the Countryside movement, accounting for 44.68% of the total sample. As nearly half of the chairpersons of the listed companies in the A-share market in the SSE and SZSE have experienced the movement, it is imperative to explore the impact of this experience on enterprises to uncover the underlying mechanism behind stock price crash risk. The changes in the proportion of *Zhiqing* over the years indicate that the number of *Zhiqing* has been decreasing. Chairpersons who grew up making outstanding contributions to nation building and the economy would eventually become advanced in age and leave the company. As they are gradually retiring from this stage of history, it is urgent to study the behavioral characteristics of this group of chairpersons with special imprints.

**TABLE 2:** Sample distribution statistics.

Year	*Zhiqing*	Non-*Zhiqing*	Total	%
2007	655	325	980	66.84
2008	672	363	1,035	64.93
2009	723	451	1,174	61.58
2010	874	625	1,499	58.31
2011	909	776	1,685	53.95
2012	942	882	1,824	51.64
2013	931	995	1,926	48.34
2014	927	1,135	2,062	44.96
2,015	936	1,336	2,272	41.20
2016	925	1,635	2,560	36.13
2017	901	1,985	2,886	31.22
2018	818	2,135	2,953	27.70
Total	10,213	12,643	22,856	44.68

Table 3 shows the descriptive statistics of the main variables. The mean and median for *NCSKEW* are −0.197 and −0.168, respectively; whereas the mean and median for *DUVOL* are −0.124 and −0.122, respectively. These findings coincide with the findings of existing research on stock price crash risk, indicating that the distribution of the explained variable in this study is reliable. The standard deviations for *NCSKEW* and *DUVOL* are 0.717 and 0.486, respectively. Such a large deviation in stock price crash risk among the sample companies is conducive to subsequent investigations. The mean for *SOE* is 0.485, indicating that the SOEs and non-SOEs are evenly distributed in the sample.

**TABLE 3:** Descriptive statistics of variables.

Variable	Obs.	Mean	Median	Std.	Min.	Max.
*NCSKEW*	17,312	–0.197	–0.168	0.717	–4.139	4.374
*DUVOL*	17,312	–0.124	–0.122	0.486	–2.313	2.534
*Zhiqing*	17,312	0.453	0.00	0.498	0.000	1.000
*Gender*	17,312	0.958	1.000	0.201	0.000	1.000
*Edu*	13,735	3.662	4.000	1.343	1.000	7.000
*Dual*	13,802	0.244	0.000	0.430	0.000	1.000
*INSIDERATE*	13,747	0.628	0.667	0.053	0.429	0.667
*TENURE*	13,489	3.497	3.611	1.012	0.693	5.094
*ABACC*	13,802	0.082	0.055	0.086	0.001	0.467
*SIGMA*	13,802	0.050	0.046	0.019	0.018	0.116
*ROA*	13,802	0.041	0.037	0.051	–0.151	0.194
*LEV*	13,802	0.437	0.433	0.212	0.051	0.912
*PPE*	13,802	0.239	0.202	0.171	0.003	0.734
*SIZE*	13,802	22.053	21.869	1.273	19.719	25.996
*MB*	13,802	2.065	1.659	1.238	0.933	7.982
*SOE*	13,650	0.485	0.000	0.500	0.000	1.000
*DTURN*	13,489	3.497	3.611	1.012	0.693	5.094
*RET*	17,312	–0.001	–0.001	0.001	–0.046	–0.000

### Univariate analysis

Table 4 exhibits the differences in *NCSKEW* and *DUVOL* between the companies with *Zhiqing* and non-*Zhiqing* chairpersons. Both *t*- and *z*-test results show that the stock price crash risk of companies with a non-*Zhiqing* chairperson is significantly higher than those with a *Zhiqing* chairperson. These results preliminarily support *H1*.

**TABLE 4:** Univariate analysis (Zhiqing vs. non-Zhiqing).

Variable	Non-Zhiqing		Zhiqing		*t*	*Wilcoxon Z*
	Mean	Median	Mean	Median		
*NCSKEW*	−0.1800805	−0.154	−0.218158	−0.186	3.4898***	3.464***
*DUVOL*	−0.1107287	−0.115	−0.1403292	−0.131	3.9924***	3.624***

***Indicate significance at the 10, 5, and 1% levels, respectively.

### Full sample multiple regression analysis

Table 5 shows the regression results for the effect of chairpersons’ Down to the Countryside experiences on stock price crash risk. Columns (1) and (2) include firm fixed effect and year fixed effect, to control for the unobserved and invariant firm and year characteristics. The regression coefficients of *Zhiqing*_*t–1*_ are −0.050 and −0.036, respectively (*p* < 0.01), indicating that the stock price crash risk of the listed companies with a *Zhiqing* chairperson is significantly lower than those with a non-*Zhiqing* chairperson. As the means for *NCSKEW* and *DUVOL* of the companies with non-*Zhiqing* chairpersons are −0.180 and −0.111, respectively, the Down to the Countryside experience can reduce the risks of stock price crashes by 27.78 and 32.43%, respectively. The economic significance of this finding is substantial. Thus, *H1* is supported. To control for the unique characteristics of different industries over time, interaction terms of the dummy variables for industry and year are included; the results are shown in Columns (3) and (4), respectively. Consistent results are observed, indicating that the findings are robust.

**TABLE 5:** Full sample regression results.

	(1)	(2)	(3)	(4)
	*NCSKEW*	*DUVOL*	*NCSKEW*	*DUVOL*
*Zhiqing*_*t–1*_	−0.050***	−0.036***	−0.051***	−0.036***
	− 2.726)	(−3.054)	(−2.744)	(−3.062)
*Gender*_*t–1*_	0.047	0.033	0.048	0.034
	(0.948)	(1.044)	(0.955)	(1.048)
*Edu*_*t–1*_	0.015**	0.010**	0.016**	0.010**
	(2.270)	(2.260)	(2.296)	(2.274)
*Age*_*t–1*_	−0.186***	−0.277***	−0.154***	−0.294***
	(−3.289)	(−4.453)	(−3.172)	(−4.893)
*DUAL*_*t–1*_	0.036*	0.009	0.036*	0.009
	(1.732)	(0.659)	(1.737)	(0.661)
*TENURE*_*t–1*_	0.009	0.008	0.009	0.008
	(0.982)	(1.361)	(0.953)	(1.345)
*INSIDERATE*_*t–1*_	−0.118	−0.094	−0.124	−0.096
	(−0.708)	(−0.881)	(−0.743)	(−0.899)
*SOE*_*t–1*_	−0.079***	−0.054***	−0.078***	−0.054***
	(−3.764)	(−3.923)	(−3.697)	(−3.887)
*ABACC*_*t–1*_	0.240**	0.119*	0.247**	0.121*
	(2.158)	(1.655)	(2.210)	(1.683)
*SIGMA*_*t–1*_	5.763***	3.680***	5.793***	3.690***
	(3.868)	(4.115)	(3.894)	(4.128)
*ROA*_*t–1*_	0.425**	0.175	0.434**	0.178
	(2.000)	(1.259)	(2.048)	(1.281)
*LEV*_*t–1*_	−0.074	−0.061	−0.072	−0.060
	(−1.299)	(−1.635)	(−1.267)	(−1.618)
*PPE*_*t–1*_	−0.044	−0.020	−0.046	−0.021
	(−0.640)	(−0.458)	(−0.669)	(−0.473)
*SIZE_*t–1*_*	0.065***	0.043***	0.066***	0.043***
	(6.438)	(6.324)	(6.518)	(6.365)
*MB*_*t–1*_	0.061***	0.045***	0.061***	0.045***
	(6.554)	(7.308)	(6.589)	(7.326)
*DTURN*_*t–1*_	−0.000	−0.000	−0.000	−0.000
	(−0.914)	(−1.210)	(−1.004)	(−1.256)
*RET*_*t–1*_	0.580***	0.460***	0.578***	0.459***
	(2.656)	(3.530)	(2.651)	(3.524)
*NCSKEW*_*t–1*_	0.055***		0.055***	
	(4.361)		(4.349)	
*DUVOL*_*t–1*_		0.028**		0.028**
		(2.310)		(2.311)
_cons	−2.057***	−1.392***	−1.241***	−1.117***
	(−7.564)	(−7.840)	(−2.639)	(−3.721)
*Firm*	Yes	Yes	Yes	Yes
*Year*	Yes	Yes	Yes	Yes
*Industry × Year*	NO	NO	Yes	Yes
*n*	8,352	8,352	8,352	8,352
Adj. *R*^2^	0.065	0.071	0.065	0.071

*, **, and *** indicate significance at the 10, 5, and 1% levels, respectively.

### Heterogeneity tests

#### Business ownership

Companies with varied business ownership generally differ greatly in the selection, motivation, and supervision of managers, and such differences are particularly prominent between SOEs and non-SOEs, which has a significant impact on the autonomy of managers’ decision-making (Jiang et al., 2009; Li et al., 2011; He and Zhang, 2015). Chairpersons of SOEs prefer to achieve political promotion and finish their term uneventfully; hence, they are vigilant regarding high-risk business decisions (Jiang et al., 2009). As such, chairpersons’ Down to the Countryside experiences may have a different effect on enterprises with different ownership types. To undercover the moderating effect of business ownership on the regression results and further distinguish the difference in the impact of chairpersons’ Down to the Countryside experiences on stock price crash risk across different ownership types, a regression analysis is conducted on a sub-sample of SOEs and non-SOEs. The results are shown in Table 6.

**TABLE 6:** Regression results by business ownership.

	SOE	Non-SOE	SOE	Non-SOE
	*NCSKEW*	*NCSKEW*	*DUVOL*	*DUVOL*
*Zhiqing*_*t–1*_	−0.004	−0.073***	−0.016	−0.048***
	(−0.134)	(−2.856)	(−0.927)	(−2.926)
*Gender*_*t–1*_	0.121	0.013	0.082*	0.007
	(1.566)	(0.209)	(1.857)	(0.175)
*Edu*_*t–1*_	0.012	0.014*	0.008	0.008*
	(0.913)	(1.695)	(0.999)	(1.653)
*Age*_*t–1*_	−0.059***	−0.128***	−0.028***	−0.097***
	(−2.987)	(−4.773)	(−3.124)	(−3.495)
*DUAL*_*t–1*_	0.036	0.022	0.002	0.003
	(0.964)	(0.862)	(0.067)	(0.204)
*TENURE*_*t–1*_	−0.003	0.013	0.002	0.007
	(−0.252)	(0.829)	(0.259)	(0.703)
*INSIDERATE*_*t–1*_	0.046	−0.148	−0.035	−0.153
	(0.194)	(−0.652)	(−0.222)	(−1.054)
*ABACC*_*t–1*_	0.255	0.133	0.143	0.016
	(1.564)	(0.852)	(1.339)	(0.164)
*SIGMA*_*t–1*_	9.293***	2.476	6.702***	1.962*
	(2.656)	(1.286)	(3.302)	(1.773)
*ROA*_*t–1*_	0.808**	0.444	0.498**	0.106
	(2.308)	(1.473)	(2.135)	(0.550)
*LEV*_*t–1*_	0.001	0.083	−0.008	−0.088*
	(0.010)	(−0.982)	(−0.146)	(−1.671)
*PPE*_*t–1*_	0.039	−0.129	0.035	−0.038
	(0.400)	(−1.234)	(0.560)	(−0.580)
*SIZE_*t–1*_*	0.049***	0.086***	0.030***	0.059***
	(3.524)	(5.132)	(3.157)	(5.677)
*MB*_*t–1*_	0.059***	0.064***	0.043***	0.044***
	(3.534)	(5.017)	(3.832)	(5.306)
*DTURN*_*t–1*_	0.000	−0.000	0.000	−0.000
	(1.557)	(−1.608)	(0.472)	(−1.334)
*RET*_*t–1*_	1.273**	0.069	1.062***	0.165
	(1.997)	(0.262)	(2.895)	(1.131)
*NCSKEW*_*t–1*_	0.024	0.070***		
	(1.226)	(4.110)		
*DUVOL*_*t–1*_			−0.012	0.054***
			(−0.683)	(3.275)
_cons	−1.509**	−1.335**	−1.227***	−1.338***
	(−2.151)	(−2.029)	(−2.973)	(−3.018)
*Firm*	Yes	Yes	Yes	Yes
*Year*	Yes	Yes	Yes	Yes
*Industry × Year*	Yes	Yes	Yes	Yes
*n*	3,779	4,714	3,779	4,714
Adj. *R*^2^	0.064	0.064	0.070	0.073
Subsample comparison of coefficients on				
*Zhiqing*	0.069 (*p* = 0.000)		0.032 (*p* = 0.000)	

*, **, and *** indicate significance at the 10, 5, and 1% levels, respectively, subsample comparison of coefficients using Fisher’s permutation is reported.

The regression coefficient of *Zhiqing*_*t–1*_ for non-SOEs (Column 2) is −0.073 (*p* < 0.01), whereas it is non-significant for SOEs (Column 1). The results demonstrate that chairpersons’ Down to the Countryside experiences have a significant effect on reducing stock price crash risk in non-SOEs, but have no effect on SOEs. The same conclusion can be reached if DUVOL is the explained variable. This finding is mainly because chairpersonship is deeply embedded in the SOE system and these chairpersons usually have government administrative titles and are subject to administrative assessment (Chang and Wong, 2009). Therefore, SOE chairpersons tend to be more cautious, and the effect of their Down to the Countryside experiences on stock price crash risk is not as significant. Non-SOEs usually aim for profit maximization, and financial performance is an important factor that may cause chairpersons to resign (Qu and Wu, 2014). To retain both their rights and positions, chairpersons of non-SOEs have greater motivation to conceal bad news, compared with their SOE counterparts, causing a rise in stock price crash risk. As such, the reducing effects of chairpersons’ Down to the Countryside experiences on stock price crash risk are greater. Hence, *H2* is rejected.

#### Further heterogeneity tests

To distinguish the effect of chairpersons’ Down to the Countryside experiences on stock price crash risk according to the retirement status of the chairperson, we divided the sample into *approaching retirement* when the chairperson’s age is greater than or equal to 59 years, and *not approaching retirement* otherwise. The regression results of retirement status are shown in Columns (1) and (2) of Table 7.

**TABLE 7:** Further heterogeneity tests.

	Approaching retirement	Not approaching retirement	Reappointed	Not reappointed	Good corporate governance	Poor corporate governance
	*NCSKEW*	*NCSKEW*	*NCSKEW*	*NCSKEW*	*NCSKEW*	*NCSKEW*
*Zhiqing*_*t–1*_	0.046	−0.074***	−0.097***	−0.048**	−0.076**	−0.022
	(0.864)	− 3.406)	(−3.207)	− 2.083)	(−2.536)	(−0.643)
*Gender*_*t–1*_	0.255**	−0.018	−0.043	0.059	−0.059	0.075
	(2.481)	(−0.339)	(−0.645)	(0.948)	(−0.729)	(0.902)
*Edu*_*t–1*_	0.000	0.005	−0.004	0.016*	0.017	0.013
	(0.016)	(0.588)	(−0.381)	(1.904)	(1.493)	(1.048)
*Age*_*t–1*_	0.495	0.002	−0.594***	−0.148*	−0.159***	−0.985***
	(1.278)	(1.459)	(−3.619)	(−2.235)	(−2.789)	(−3.159)
*DUAL*_*t–1*_	−0.015	0.036	−0.020	0.053**	0.017	0.046
	(−0.317)	(1.572)	(−0.515)	(2.132)	(0.525)	(1.219)
*TENURE*_*t–1*_	−0.011	0.014	−0.003	0.017	0.013	−0.007
	(−0.467)	(1.304)	(−0.194)	(0.952)	(0.815)	(−0.441)
*INSIDERATE*_*t–1*_	−0.689**	−0.040	−0.334	−0.058	−0.049	−0.398
	(−2.144)	(−0.209)	(−1.239)	(−0.281)	(−0.158)	(−1.358)
*SOE*_*t–1*_	−0.051	−0.083***	−0.031	−0.096***	−0.036	−0.094**
	(−1.123)	(−3.339)	(−0.836)	(−3.614)	(−1.035)	(−2.344)
*ABACC*_*t–1*_	0.335	0.232*	0.143	0.384***	0.015	0.104
	(1.538)	(1.832)	(0.805)	(2.666)	(0.084)	(0.568)
*SIGMA*_*t–1*_	2.517	6.727***	8.296***	5.376***	5.964***	5.574
	(0.728)	(4.119)	(2.773)	(2.955)	(2.641)	(1.577)
*ROA*_*t–1*_	0.050	0.583**	0.327	0.578**	0.717*	1.008***
	(0.113)	(2.487)	(1.009)	(2.158)	(1.880)	(2.721)
*LEV*_*t–1*_	−0.008	−0.087	−0.076	−0.074	−0.093	−0.040
	(−0.053)	(−1.379)	(−0.827)	(−1.005)	(−0.921)	(−0.417)
*PPE*_*t–1*_	−0.153	−0.045	0.058	−0.147	−0.154	−0.179
	(−1.035)	(−0.565)	(0.511)	(−1.630)	(−1.221)	(−1.423)
*SIZE_*t–1*_*	0.051**	0.062***	0.070***	0.060***	0.046***	0.058***
	(2.330)	(5.324)	(4.379)	(4.649)	(2.844)	(3.187)
*MB*_*t–1*_	0.078***	0.054***	0.047***	0.067***	0.053***	0.053***
	(3.769)	(5.134)	(3.145)	(5.521)	(3.551)	(3.097)
*DTURN*_*t–1*_	−0.000	−0.000	−0.000*	-0.000	−0.000	−0.000
	(−0.413)	(−0.658)	(−1.792)	(-0.004)	(−0.672)	(−1.197)
*RET*_*t–1*_	0.114	0.686***	0.947**	0.509*	0.369	0.368
	(0.224)	(2.911)	(2.095)	(1.929)	(1.259)	(0.628)
*NCSKEW*_*t–1*_	0.053**	0.058***	0.075***	0.051***	0.074***	0.047**
	(2.183)	(3.821)	(3.255)	(3.454)	(3.683)	(2.007)
_cons	−0.816	−1.184**	−1.250*	-1.462**	−0.016	−1.882**
	(−0.693)	(−2.235)	(−1.648)	(-2.272)	(−0.020)	(−2.336)
*Firm*	Yes	Yes	Yes	Yes	Yes	Yes
*Year*	Yes	Yes	Yes	Yes	Yes	Yes
*Industry × Year*	Yes	Yes	Yes	Yes	Yes	Yes
*n*	1,855	6,518	2,794	5,579	3,040	2,958
Adj. *R*^2^	0.059	0.068	0.059	0.070	0.097	0.054
**Subsample comparison of coefficients on**						
*Zhiqing*	0.12 (*p* = 0.000)		−0.049 (*p* = 0.000)		−0.054 (*p* = 0.000)	

*, **, and *** indicate significance at the 10, 5, and 1% levels, respectively, subsample comparison of coefficients using Fisher’s permutation is reported.

The results indicate that the Down to the Countryside experiences of chairpersons approaching retirement do not reduce stock price crash risk. This could be owed to the fact that chairpersons approaching retirement prefer to maintain the status quo and transit uneventfully into retirement; hence, the effect of their Down to the Countryside experiences is not significant. However, the effect of experiences on stock price crash risk is significant among chairpersons not approaching retirement. The same results can be obtained if the dependent variable is *DUVOL*. Hence, *H3* is supported. This finding shows that more attention should be paid to the role of the personal experiences of chairpersons not approaching retirement.

The likelihood of being re-appointed was expected to moderate the influence of experiences on stock price crash risk. The single-term tenure of a chairperson in China usually does not exceed three years. Therefore, we partitioned the sample into *reappointment* (tenure ≥ 36 months) and *not reappointment* (tenure < 36 months). As shown in Columns (3) and (4) of Table 7, regardless of reappointment, chairpersons’ Down to the Countryside experiences significantly reduce stock price crash risk; however, this effect is more prominent among re-appointed chairpersons. We found the same results in regression results with *DUVOL* as the dependent variable. Hence, *H4* is supported.

Following Bai et al. (2004), we used the balance of shareholder power as a proxy variable for internal governance. We divided the sample into *good corporate governance* and *poor corporate governance*, depending on whether the ratio of the second to fifth largest shareholders to the first largest shareholder was greater than the industry median value. As shown in Columns (5) and (6) of Table 7, the effect of chairpersons’ Down to the Countryside experiences on reducing stock price crash risk is greater in companies with good internal governance. The same results apply when *DUVOL* was utilized as the dependent variable. Hence, *H5* is supported. Therefore, companies with strong governance policies should fully utilize the effects of their policy to ensure their development trajectory.

### Alleviating potential endogeneity

#### Controlling self-selection bias

The research design may be susceptible to a self-selection bias. Companies in certain industries, like agriculture, might be more inclined to hire a chairperson with Down to the Countryside experiences, as they have more agricultural experiences. The most commonly used methods for correcting self-selection bias are the two-stage switching regression analysis (Heckman, 1979) and the two-stage treatment effect model (Maddala, 1983). As *Zhiqing* was a dummy variable, we selected the treatment effect model to control any self-selection bias.

The data were sorted by industry-year, and the means for *Zhiqing* by industry and year (*Zhiqingmean*) were calculated and used as an exogenous variable representing the industry’s tendency to hire chairpersons with Down to the Countryside experiences. Maddala (1983) two-stage model was applied:

Stage 1


$$Pr\left(Zhiqing_{i,t-1}=1\right)=\beta_{0}+\beta_{1}\times Size_{i,t-1}$$

$$+\beta_{2}\times R\,o\,a_{i,t-1}+\beta_{3}\times L\,e\,v_{i,t-1}+\beta_{4}\times R\,E\,T_{i,t-1}$$

$$+\beta_{5}\times M\,B_{i,t-1}+\beta_{6}\times P\,P\,E_{i,t-1}+\beta_{7}$$

(6) $$\times Z\,h\,i\,q\,i\,n\,g\,m\,e\,a\,n_{j,t-1}\,Y\,e\,a\,r+\varepsilon_{i,t-1},$$

Stage 2


(7) $$N\,C\,S\,K\,E\,W_{i,t}=\beta_{0}+\beta_{1}\times Z\,h\,i\,q\,i\,n\,g_{i,t-1}+\beta_{i}$$

$$\times C\,o\,n\,t\,r\,o\,l\,s_{i,t-1}+\beta_{j}\times \hat{l\,a\,m\,b\,d\,a}+\varepsilon_{i,t},$$

(8) $$D\,U\,V\,O\,L_{i,t}=\beta_{0}+\beta_{1}\times Z\,h\,i\,q\,i\,n\,g_{i,t-1}+\beta_{i}$$

$$\times C\,o\,n\,t\,r\,o\,l\,s_{i,t-1}+\beta_{j}\times \hat{l\,a\,m\,b\,d\,a}+\varepsilon_{i,t}.$$

Table 8 displays the results. The regression coefficients of *lambda* are all significantly positive, indicating that there is a self-selection bias in the baseline regression, and results in a correction for self-selection bias. After controlling for other variables, the regression coefficients of *Zhiqing*_*t–1*_ for NCSKEW and DUVOL are −0.227 and −0.183, respectively (*p* < 0.01). These findings demonstrate that, following the correction for the self-selection bias, chairpersons’ Down to the Countryside experiences still reduce stock price crash risk. Additionally, according to the untabulated first-stage regression results, *Zhiqing*_*t–1*_ is significantly affected by *Zhiqingmean*, verifying that the industry tendency influences an individual firm’s decision on appointment.

**TABLE 8:** Empirical results of the two-stage treatment effect model.

	*NCSKEW*	*DUVOL*
*Zhiqing*_*t–1*_	−0.227***	−0.183***
	(−3.69)	(−4.67)
*Gender*_*t–1*_	0.013	0.010
	(0.30)	(0.35)
*Edu*_*t–1*_	0.009	0.006
	(1.32)	(1.48)
*Age*_*t–1*_	−0.128**	−0.388**
	(−2.08)	(−2.49)
*DUAL*_*t–1*_	0.033	0.009
	(1.61)	(0.66)
*TENURE*_*t–1*_	−0.001	0.001
	(−0.10)	(0.18)
*INSIDERATE*_*t–1*_	−0.102	−0.068
	(−0.63)	(−0.67)
*SOE*_*t–1*_	−0.099***	−0.063***
	(−4.79)	(−4.82)
*ABACC*_*t–1*_	0.029	−0.026
	(0.28)	(−0.39)
*SIGMA*_*t–1*_	7.876***	4.668***
	(3.98)	(3.70)
*ROA*_*t–1*_	0.712***	0.378***
	(3.66)	(3.03)
*LEV*_*t–1*_	−0.006	−0.028
	(−0.10)	(−1.35)
*PPE*_*t–1*_	−0.023	−0.001
	(−0.39)	(−0.80)
*SIZE_*t–1*_*	0.051***	0.037***
	(5.49)	(6.27)
*MB*_*t–1*_	0.066***	0.047***
	(7.81)	(8.72)
*DTURN*_*t–1*_	−0.000***	−0.000***
	(−5.70)	(−6.24)
*RET*_*t–1*_	1.620***	1.092***
	(4.99)	(5.28)
*NCSKEW*_*t–1*_	0.059***	
	(6.72)	
*DUVOL*_*t–1*_		0.034***
		(2.97)
Lambda	0.104***	0.087***
	(12.27)	(3.49)
*Constant*	−1.689***	−1.176***
	(−6.72)	(−7.32)
*Firm*	Yes	Yes
*Year*	Yes	Yes
*n*	8,373	8,376
χ^2^	436.651	460.608

** and *** indicate significance at the 10, 5, and 1% levels, respectively. Results from the first stage are untabulated for brevity.

#### Difference-in-differences (DID)

Some company-level factors, such as corporate culture and goodwill, may affect stock price crash risk. However, such factors are usually difficult to measure, resulting in endogeneity problems. In this study, change in chairpersons is introduced as an exogenous event for a DID test. A new between-group dummy variable (*New*_*i,t*_) was used. If a non-*Zhiqing* chairperson was replaced by a *Zhiqing* chairperson, the value was 1, and if neither chairperson have Down to the Countryside experience, the value was 0. As the change in stock price crash risk caused by the change in chairpersons tends to be lagged, a narrow time window was not conducive to observing the impact. Therefore, this study introduced a variable (*Post*_*i,t*_) to indicate time. *Post*_*i,t*_ of the year when the chairperson was changed and the following 2 years = 1, and *Post*_*i,t*_ of the three years prior to the change = 0. For robustness, a dummy variable *New_N_*i,t*_* was introduced and set up in the opposite way as *New*_*i,t*_. If a *Zhiqing* chairperson was replaced by a non-*Zhiqing* chairperson, the value was 1; otherwise, the value was 0. The values assigned to *Post*_*i,t*_ remained the same as mentioned above. If the coefficient of *New_*i,t*_ × Post_*i,t*_* (*New_N_*i,t*_ × Post_*i,t*_*) was significantly positive (negative), then it confirmed that the chairpersons’ Down to the Countryside experiences significantly reduce stock price crash risk. The DID models were constructed based on Equations (9) and (10), and the results are presented in Table 9.


(9) $$C\,R\,A\,S\,H\,R\,I\,S\,K_{i,t}=\beta_{0}+\beta_{1}\times N\,e\,w_{i,t}\times P\,o\,s\,t_{i,t}$$

$$+\beta_{i}\times C\,o\,n\,t\,r\,o\,l\,s_{i,t}+F\,i\,x\,e\,d\,e\,f\,f\,e\,c\,t\,s+\varepsilon_{i,t}$$

(10) $$C\,R\,A\,S\,H\,R\,I\,S\,K_{i,t}=\beta_{0}+\beta_{1}\times N\,e\,w\,\text{\_}\,N_{i,t}\times P\,o\,s\,t_{i,t}$$

$$+\beta_{i}\times C\,o\,n\,t\,r\,o\,l\,s_{i,t}+F\,i\,x\,e\,d\,e\,f\,f\,e\,c\,t\,s+\varepsilon_{i,t}$$

**TABLE 9:** Difference-in-differences results.

	Non-Zhiqing replaced by Zhiqing		Zhiqing replaced by non-Zhiqing	
	*NCSKEW*	*DUVOL*	*NCSKEW*	*DUVOL*
*New* × *Post*	−0.210**	−0.103**		
	(−2.221)	(−2.158)		
*New_N* × *Post*			0.167*	0.113**
			(1.882)	(2.147)
*Gender*	−0.109	−0.076	0.017	0.089
	(−0.997)	(−1.635)	(0.982)	(1.524)
*Edu*	−0.686	−0.243*	0.489	0.187
	(−0.895)	(−1.875)	(1.350)	(0.986)
*Age*	−0.003	−0.005	0.004	0.004
	(−0.368)	(−1.280)	(0.708)	(1.359)
*Dual*	0.208	0.143*	−0.029	0.030
	(1.342)	(1.762)	(−0.390)	(0.688)
*TENURE*	−0.025	−0.017	0.017	−0.005
	(−0.440)	(−0.535)	(0.575)	(−0.303)
*INSIDERATE*	0.984	−0.040	0.357	0.172
	(0.945)	(−0.062)	(0.787)	(0.562)
*SOE*	−0.039	−0.167**	−0.224***	−0.112**
	(−0.309)	(−2.360)	(−3.566)	(−2.563)
*ABACC*	−0.220	−0.002	−0.693**	−0.297
	(−0.549)	(−0.011)	(−2.006)	(−1.281)
*SIGMA*	11.098	11.696*	5.881	3.762
	(0.915)	(1.849)	(0.984)	(1.009)
*DTURN*	−0.000*	−0.000*	−0.000	−0.000
	(−2.007)	(−2.573)	(−1.023)	(−1.262)
*ROA*	−0.109	0.442	−0.116	−0.267
	(−0.174)	(0.779)	(−0.250)	(−0.784)
*LEV*	0.324	−0.102	0.056	0.053
	(1.168)	(−0.694)	(0.384)	(0.541)
*PPE*	−0.321	−0.015	−0.139	−0.126
	(−0.824)	(−0.086)	(−1.057)	(−1.433)
*SIZE*	0.067	0.040	0.052**	−0.001
	(1.176)	(1.070)	(2.134)	(−0.073)
*MB*	0.023	0.019	0.068***	0.031*
	(0.383)	(0.685)	(2.827)	(1.875)
*DTURN*	−0.000	−0.000	−0.000	−0.000
	(−0.965)	(−1.003)	(−1.058)	(−0.488)
*RET*	2.073	2.099**	1.574	1.112*
	(1.091)	(2.145)	(1.597)	(1.786)
*NCSKEW*	0.034***		0.075***	
	(2.972)		(3.586)	
*DUVOL*		0.042***		0.042***
		(3.533)		(4.557)
_*cons*	−2.532	−0.929	−2.218***	−0.609
	(−1.553)	(−0.807)	(−3.067)	(−1.379)
*Firm*	Yes	Yes	Yes	Yes
*Year*	Yes	Yes	Yes	Yes
*n*	221	221	790	790
*R* ^2^	0.124	0.141	0.055	0.049

*, **, and *** indicate significance at the 10, 5, and 1% levels, respectively.

When non-*Zhiqing* chairpersons are replaced by *Zhiqing* chairpersons, the coefficients of *New × Post* are −0.210 (*p* = 0.027) and −0.103 (*p* = 0.032). When *Zhiqing* chairpersons are replaced by non-*Zhiqing* chairpersons, the coefficients of *New_N × Post* are 0.167 (*p* = 0.060) and 0.113 (*p* = 0.032), respectively. These findings indicate that the shift from non-*Zhiqing* chairpersons to *Zhiqing* chairpersons decreases stock price crash risk, while the shift from *Zhiqing* chairpersons to non-*Zhiqing* chairpersons results in the opposite; hence, the change in *Zhiqing* chairpersons significantly reduces stock price crash risk. The two DID results further confirm the findings that Down to the Countryside experiences significantly reduce stock price crash risk. These findings provide empirical evidence regarding the impact of chairpersons’ early life experiences on their current management of companies.

### Robustness check

#### Re-measuring the Down to the Countryside experience

Although being sent to the countryside was compulsory at the time, individuals could be exempt for certain reasons, such as being in the military, experiencing a severe illness, or not being currently in mainland China. Hence, the original categorization criteria do not apply to all individuals, which could affect the accuracy of the conclusions. Therefore, we further screened the chairpersons based on information obtained from Baidu Baike, the Wind Economic Database, the chairpersons’ personal profiles, talk show interviews, and memoirs. Elimination and keyword searches were used to redefine *Zhiqing*. The detailed methods are presented in Panel A of Table 10.

**TABLE 10:** Robustness check methods and results.

Method		Criteria used to re-categorize *Zhiqing*		How the samples are adjusted		
**Panel A: Methods used to re-test the Down to the Countryside effects**						
Elimination method		Remove the individuals who are known to be in the military, ill, or outside of mainland China from December 1968 to December 1977 from the *Zhiqing* sample (1) to the non-*Zhiqing* sample (0).		176 observations were excluded.		
Keyword method		Chairpersons with related terms in their personal profile, such as “z*hiqing* (educated youth),” “*nongchang* (farm),” “*jieshou zaijiaoyu* (receiving re-education),” “*chadui* (Down to the Countryside)” “*xiaxiang laodong* (work in the countryside),” “*shangshan xiaxiang* (Up to the Mountains and Down to the Countryside movement),” and “*shengchan jianshe bingtuan* (production and construction corps)” are assigned the value of 1 for *Zhiqing*; otherwise, the value is 0.		279 *Zhiqing* observations were retained.		

	**Elimination method**		**Keyword method**		**“Lao san jie” observations excluded**	
			
	***NCSKEW***	***DUVOL***	***NCSKEW***	***DUVOL***	***NCSKEW***	***DUVOL***

**Panel B: Regression results following adjustment**						
*Zhiqing*_*t–1*_	−0.063***	−0.045***	−0.071***	−0.049***	−0.053***	−0.037***
	(−3.441)	(−3.876)	(−2.910)	(−3.110)	(−2.812)	(−3.083)
*Gender*_*t–1*_	0.026	0.019	0.047	0.056	0.047	0.031
	(0.523)	(0.591)	(0.744)	(1.335)	(0.931)	(1.003)
*Edu*_*t–1*_	0.009	0.006	0.011	0.007	0.017**	0.011**
	(1.338)	(1.454)	(1.250)	(1.171)	(2.504)	(2.587)
*Age*_*t–1*_	−0.189***	−0.235***	−0.244**	−0.327***	−0.182***	−0.129***
	(−3.163)	(−3.634)	(−2.319)	(−4.425)	(−4.869)	(−3.541)
*DUAL*_*t–1*_	0.032	0.008	0.030	0.001	0.037*	0.011
	(1.557)	(0.578)	(1.097)	(0.041)	(1.747)	(0.848)
*TENURE*_*t–1*_	0.012	0.009	0.013	0.018**	0.009	0.007
	(1.253)	(1.523)	(1.028)	(2.219)	(0.905)	(1.106)
*INSIDERATE*_*t–1*_	−0.149	−0.109	−0.035	−0.048	−0.120	−0.094
	(−0.902)	(−1.033)	(−0.157)	(−0.332)	(−0.712)	(−0.905)
*SOE*_*t–1*_	−0.075***	−0.052***	−0.024	−0.019	−0.074***	−0.050***
	(−3.557)	(−3.818)	(−0.824)	(−0.984)	(−3.422)	(−3.671)
*ABACC*_*t–1*_	0.261**	0.126*	0.024	0.030	0.243**	0.095
	(2.338)	(1.764)	(0.174)	(0.334)	(2.130)	(1.336)
*SIGMA*_*t–1*_	5.826***	3.788***	12.896**	10.457***	8.179***	5.750***
	(3.933)	(4.266)	(2.294)	(2.779)	(3.874)	(4.403)
*ROA*_*t–1*_	0.494**	0.208	0.698***	0.464**	0.440**	0.199
	(2.353)	(1.519)	(2.617)	(2.505)	(2.054)	(1.518)
*LEV*_*t–1*_	−0.072	−0.060	−0.026	−0.025	−0.079	−0.063*
	(−1.283)	(−1.638)	(−0.339)	(−0.495)	(−1.366)	(−1.723)
*PPE*_*t–1*_	−0.068	−0.036	−0.091	−0.036	−0.048	−0.016
	(−0.986)	(−0.817)	(−0.950)	(−0.605)	(−0.684)	(−0.361)
*SIZE_*t–1*_*	0.062***	0.041***	0.044***	0.028***	0.067***	0.043***
	(6.196)	(6.161)	(3.438)	(3.386)	(6.513)	(6.401)
*MB*_*t–1*_	0.058***	0.043***	0.060***	0.045***	0.060***	0.042***
	(6.359)	(7.206)	(4.413)	(4.985)	(6.332)	(7.063)
*DTURN*_*t–1*_	−0.000	−0.000	−0.000	−0.000	−0.000	−0.000
	(−0.814)	(−1.067)	(−0.027)	(−0.454)	(−1.024)	(−0.984)
*RET*_*t–1*_	0.568***	0.459***	1.689	1.572**	1.008***	0.843***
	(2.617)	(3.541)	(1.517)	(2.079)	(2.965)	(3.984)
*NCSKEW*_*t–1*_	0.063***		0.063***		0.056***	
	(4.950)		(3.171)		(4.29)	
*DUVOL*_*t–1*_		0.037***		0.035**		0.028**
		(3.055)		(2.008)		(2.30)
*Firm*	Yes	Yes	Yes	Yes	Yes	Yes
*Year*	Yes	Yes	Yes	Yes	Yes	Yes
*Industry × Year*	Yes	Yes	Yes	Yes	Yes	Yes
*n*	8,373	8,373	407	407	8,098	8,098
Adj. *R*^2^	0.068	0.073	0.068	0.067	0.066	0.071

*, **, and *** indicate significance at the 10, 5, and 1% levels, respectively.

This elimination method excluded chairpersons known not to have participated in the movement. Searching terms related to the movement, such as “educated youth” and “farm,” were then compiled from personal profiles. Chairpersons who were identified using these search terms were assumed to have experienced a direct psychological impact due to the movement; hence, it was expected that these chairpersons are more likely to be influenced by the experience and more cautious when making decisions, thereby reducing stock price crash risk. Therefore, these observations were analyzed separately. As the sample size following the search was small, which could affect the accuracy of the regression analysis, a propensity score matching was adopted to restructure the sample prior to performing the regression analysis. The results are shown in Panel B of Table 10. The coefficients of *Zhiqing*_*t–1*_ are negative on both occasions (*p* < 0.01). Additionally, the adjusted coefficients of the sample screened through the elimination method are −0.063 and −0.045, respectively, which are lower than that of the baseline results (−0.051 and −0.036, respectively). These findings demonstrate that the elimination method is effective. The coefficients of *Zhiqing*_*t–1*_ following the keyword search process are −0.071 and −0.049, respectively, which are lower than the coefficients obtained following the elimination method. These findings indicate that chairpersons who are involved in the Down to the Countryside experience in their personal profiles have a stronger connection to the experience and the experience has a more profound influence on them. Therefore, compared with chairpersons without Down to the Countryside experience, these chairpersons tend to possess more movement-shaped personal characteristics, making them more cautious, which reduces stock price crash risk.

As mentioned above, the policy was implemented in 1968, and the students who were first sent to the countryside were 1966, 1967, and 1968 graduates (known as the “*lao san jie*”). Being the first group of students sent to the countryside, they experienced more difficulties and were more likely to develop the distinct characteristics outlined above, compared with students who were sent down to the countryside later. To further examine the robustness of the findings, the “*lao san jie*” observations are excluded. Specifically, as the oldest students who graduated in 1969 were 19 years old, chairpersons aged between 71 and 73 years are excluded from the regression analysis. As shown in Panel B of Table 10, the coefficients of *Zhiqing*_*t–1*_ for *NCSKEW* and *DUVOL* are still significant (*p* < 0.01), indicating that the results remain robust following the removal of “*lao san jie*” observations.

#### Placebo test

**FIGURE 1:**
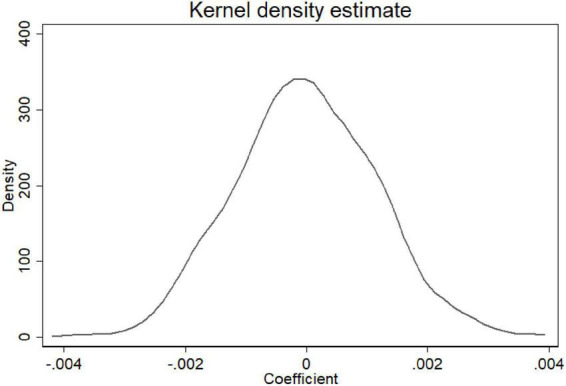
Kernel density plot for the placebo test.

To reduce the bias caused by the contingency of the definition of *Zhiqing* and any omitted-variable bias, this study adopted a placebo test, in which chairpersons were randomly assigned to *Zhiqing.* The generated dummy variable was used to replace the original variable for the regression analysis. If the results were not significant, then the original definition of *Zhiqing* is satisfactory, and the original findings were maintained. To reduce the effects of randomness, 1,000 draws were performed, and the results were used to generate a kernel density estimation plot. The plot is shown in Figure 1. The kernel density plot presents a quasi-normal distribution, and the peak is located approximately around 0. These results indicate that, following random assignment, the coefficients do not manifest a particular characteristic; hence, the original definition of *Zhiqing* is reasonable, and the original findings are considered valid.

### Further investigation

#### Underlying mechanism

Chen et al. (2021) argued that CEOs who experienced early-life disasters are more risk tolerant, and thus more willing to accept the risks associated with bad news hoarding, engendering the formation of stock price crashes. Chen et al. (2019) argued that labor unions are able to lower the probability of stock price crash risk by reducing managerial risk-taking behaviors. It is assumed that chairpersons with Down to the Countryside experience tend to be more conservative when making risky decisions, compared with those without such experience. Therefore, this section investigates chairpersons’ propensity to risk and explores the underlying mechanisms of the impact of their experience on stock price crash risk. Referring to the methods proposed by Faccio et al. (2011) and Song et al. (2018), the calculation results are expressed as percentages to obtain the risk-taking variables (*Risk1* and *Risk2*).


(11) $$A\,d\,j\,\_\,R\,o\,a_{i,t}=\frac{E\,B\,I\,T_{i,t}}{A\,S\,S\,E\,T_{i,t}}-\frac{1}{X}\,\sum_{k=1}^{X}\frac{E\,B\,I\,T_{i,t}}{A\,S\,S\,E\,T_{i,t}},$$

$$Risk1_{i,t}=\sqrt{\frac{1}{T-1}\sum_{t=1}^{T}{\left(Adj\_Roa_{i,t}-\frac{1}{T}\sum_{t=1}^{T}Adj\_Roa_{i,t}\right)}^{2}|T=3,}$$

(13) R⁢i⁢s⁢k⁢2i,t=M⁢a⁢x⁢(A⁢d⁢j⁢_⁢R⁢o⁢ai,t)-M⁢i⁢n⁢(A⁢d⁢jR⁢o⁢ai,t).

*Risk1*, *Risk2*, and the interaction term between *Risk* and *Zhiqing* are added to the regression model (Equation 14), and the results are shown in Table 11.


(14) $$C\,R\,A\,S\,H\,R\,I\,S\,K_{i,t}=\beta_{0}+\beta_{1}\times Z\,h\,i\,q\,i\,n\,g_{i,t-1}$$

$$+\beta_{2}\times R\,i\,s\,k_{i,t-1}+\beta_{3}\times Z\,h\,i\,q\,i\,n\,g_{t-1}\times R_{t-1}$$

$$+\beta_{i}\times C\,o\,n\,t\,r\,o\,l\,s+F\,i\,x\,e\,d\,e\,f\,f\,e\,c\,t\,s+\varepsilon_{i,t}$$

**TABLE 11:** Underlying mechanism test results.

	*Risk1*		*Risk2*	
	*NCSKEW*	*DUVOL*	*NCSKEW*	*DUVOL*
*Zhiqing*_*t–1*_	−0.014	−0.015	−0.013	−0.014
	(−0.586)	(−0.910)	(−0.543)	(−0.883)
*Risk*_*t–1*_	1.385***	0.495	0.728***	0.254
	(2.687)	(1.348)	(2.675)	(1.312)
*Zhiqing*_*t–1*_ × *R*_*t–1*_	−1.595**	−0.817*	−0.868**	−0.442*
	(−2.245)	(−1.721)	(−2.308)	(−1.769)
*Gender*_*t–1*_	0.055	0.042	0.055	0.042
	(0.896)	(1.104)	(0.894)	(1.103)
*Edu*_*t–1*_	0.009	0.005	0.009	0.005
	(1.183)	(1.092)	(1.185)	(1.093)
*Age*_*t–1*_	−0.210***	−0.198***	−0.317***	−0.282***
	(−3.223)	(−2.802)	(−3.441)	(−3.294)
*DUAL*_*t–1*_	0.049**	0.010	0.049**	0.010
	(2.152)	(0.659)	(2.147)	(0.656)
*TENURE*_*t–1*_	0.012	0.012*	0.012	0.012*
	(1.176)	(1.735)	(1.177)	(1.732)
*INSIDERATE*_*t–1*_	−0.037	0.018	−0.038	0.018
	(−0.204)	(0.151)	(−0.208)	(0.148)
*SOE*_*t–1*_	−0.028	−0.019	−0.028	−0.019
	(−1.175)	(−1.218)	(−1.178)	(−1.223)
*ABACC*_*t–1*_	0.101	0.076	0.102	0.077
	(0.866)	(0.965)	(0.873)	(0.974)
*SIGMA*_*t–1*_	8.138***	5.263***	8.140***	5.265***
	(4.840)	(5.254)	(4.842)	(5.257)
*ROA*_*t–1*_	0.674***	0.333**	0.669***	0.331**
	(3.020)	(2.181)	(2.999)	(2.164)
*LEV*_*t–1*_	−0.002	−0.024	−0.002	−0.024
	(−0.033)	(−0.556)	(−0.031)	(−0.552)
*PPE*_*t–1*_	−0.019	−0.018	−0.019	−0.018
	(−0.250)	(−0.360)	(−0.243)	(−0.352)
*SIZE_*t–1*_*	0.032***	0.022***	0.032***	0.022***
	(2.894)	(3.000)	(2.887)	(2.992)
*MB*_*t–1*_	0.034***	0.030***	0.034***	0.030***
	(3.420)	(4.576)	(3.428)	(4.591)
*DTURN*_*t–1*_	−0.000	−0.000	−0.000	−0.000
	(−1.414)	(−1.547)	(−1.411)	(−1.545)
*RET*_*t–1*_	0.695***	0.527***	0.695***	0.527***
	(3.016)	(3.936)	(3.018)	(3.939)
*NCSKEW*_*t–1*_	0.059***		0.059***	
	(3.892)		(3.894)	
*DUVOL*_*t–1*_		0.040***		0.040***
		(2.851)		(2.852)
_cons	−0.536	−0.592	−0.537	−0.591
	(−0.827)	(−1.421)	(−0.828)	(−1.419)
*Firm*	Yes	Yes	Yes	Yes
*Year*	Yes	Yes	Yes	Yes
*Industry × Year*	Yes	Yes	Yes	Yes
*n*	6,097	6,097	6,097	6,097
Adj. *R*^2^	0.055	0.052	0.055	0.052

*, **, and *** indicate significance at the 10, 5, and 1% levels, respectively.

With *NCSKEW* as the explained variable and after controlling for other variables, the regression coefficients of risk-taking are significantly positive in both calculation methods. These results coincide with those of Sun et al. (2018), who revealed that a rise in risk-taking ability significantly increases stock price crash risk. With the explained variable as *DUVOL*, the regression coefficients of *Risk* are both positive; however, the results are not significant. Additionally, the coefficients of *ZR* are negative and significant in both calculation methods and for both explained variables, indicating that chairpersons’ Down to the Countryside experiences significantly reduce companies’ risk-taking and stock price crash risk. As such, the most likely influence channel that explains these results is Down to the Countryside experience → risk-taking ability → stock price crash risk.

#### Economic consequences

Lastly, the economic consequences of the impact of chairpersons’ Down to the Countryside experience on stock price crash risk were examined. Firm value was used as a proxy for economic consequences. Based on Wang et al. (2019), this study used Tobin’s Q (*TobinQ*) to measure firm value. Along with the control variables in the baseline regression, other control variables that influence firm value, such as the number of board meetings held (*Meeting*), board size (*Bsize*), firm growth (*Growth*), firm age (*firmage*), and operating cash flow (Ocf) were applied. Considering the likelihood of lagged effects, Tobin’s Q of 1 to 3 *lead* (*t+1*, *t+2*, and *t+3*) were used as the explained variables. The model was constructed according to Equation (15).


(15) $$T\,o\,b\,i\,n\,Q_{i,t+1,2,3}=\beta_{0}+\beta_{1}\times Z\,h\,i\,q\,i\,n\,g_{i,t}$$

$$\times C\,R\,A\,S\,H\,R\,I\,S\,K_{i,t}+\beta_{2}\times Z\,h\,i\,q\,i\,n\,g_{i,t}+\beta_{3}$$

$$\times C\,R\,A\,S\,H\,R\,I\,S\,K_{i,t}+\beta_{i}\times C\,o\,n\,t\,r\,o\,l\,s+F\,i\,x\,e\,d\,e\,f\,f\,e\,c\,t\,s+\varepsilon_{i,t}.$$

The results are shown in Table 12. The coefficients of the interaction term between *Zhiqing* and *CRASHRISK* are positive and statistically significant for the 1 and 2 *lead* of both *NCSKEW* and *DUVOL*. This finding indicates that chairpersons’ Down to the Countryside experiences promote firm value by reducing stock price crash risk. However, when the explained variable is *TobinQ*_t+3_, the coefficients are negative but not significant. Additionally, the regression coefficients of the interaction term between *Zhiqing* and *CRASHRISK* for *Tobinq*_*t+2*_ are smaller than those for *TobinQ*_*t+1*_. These findings indicate that the impact of Down to the Countryside experiences on firm value through reducing stock price crash risk wane over time and cease to exist at *t+3*. As such, the experience has an impact on enhancing firm value through reducing stock price crash risk over the short term; however, the effect tends to decline over time. These findings may provide a new perspective during recruitment when evaluating chairpersons’ personal experiences and defining their tenure.

**TABLE 12:** Influence of Down to the Countryside experiences on firm value.

	*NCSKEW*			*DUVOL*		
	*Tobinq*_*t+1*_	*Tobinq*_*t+2*_	*Tobinq*_*t+3*_	*Tobinq*_*t+1*_	*Tobinq*_*t+2*_	*Tobinq*_*t+3*_
*Zhiqing* × *Crashrisk*	0.215***	0.166**	−0.019	0.309**	0.277***	−0.014
	(2.735)	(2.379)	(−0.202)	(2.519)	(2.584)	(−0.096)
*Zhiqing*	0.084	0.106	0.062	0.088	0.122	0.065
	(0.960)	(1.184)	(0.603)	(0.996)	(1.319)	(0.613)
*NCSKEW*	−0.044	−0.053	0.096			
	(−0.635)	(−0.860)	(1.088)			
*DUVOL*				−0.081	−0.095	0.115
				(−0.787)	(−1.074)	(0.920)
*Gender*_*t–1*_	1.267	1.142	−0.265	0.165	1.321	−1.118
	(0.254)	(0.842)	(−0.257)	(1.487)	(1.032)	(−1.189)
*Edu*_*t–1*_	0.333**	0.669	0.331	−0.161	−0.091	−0.034
	(2.43)	(0.999)	(0.694)	(−0.947)	(−0.647)	(−1.39)
*Age*_*t–1*_	−0.024***	−0.002*	−0.024	0.259***	0.236	0.196
	(−3.204)	(−1.892)	(−0.557)	(3.648)	(0.916)	(1.361)
*Dual*	0.095	0.015	0.028	0.097	0.017	0.030
	(0.940)	(0.155)	(0.261)	(0.960)	(0.174)	(0.280)
*INSIDERATE*	−1.053	−1.544**	−2.181***	−1.046	−1.538**	−2.176***
	(−1.593)	(−2.404)	(−3.080)	(−1.587)	(−2.402)	(−3.076)
*Bsize*	−0.015	−0.014	−0.027	−0.015	−0.015	−0.027
	(−0.667)	(−0.644)	(−1.177)	(−0.693)	(−0.663)	(−1.173)
*Meeting*	0.260***	0.258***	0.094	0.259***	0.256***	0.096
	(2.656)	(2.920)	(1.043)	(2.648)	(2.916)	(1.065)
*LEV*	−0.907***	−0.738***	−0.218	−0.903***	−0.733***	−0.218
	(−3.243)	(−2.628)	(−0.607)	(−3.235)	(−2.617)	(−0.609)
*SIZE*	−0.397***	−0.479***	−0.513***	−0.396***	−0.479***	−0.512***
	(−9.096)	(−9.454)	(−8.845)	(−9.098)	(−9.468)	− 8.847)
*Ocf*	1.438***	2.279***	2.110***	1.448***	2.284***	2.118***
	(2.956)	(4.411)	(4.160)	(2.977)	(4.430)	(4.178)
*Companyage*	−0.030***	−0.014*	−0.012	−0.030***	−0.014*	−0.012
	(−3.889)	(−1.874)	(−1.507)	(−3.870)	(−1.856)	(−1.490)
*Growth*	0.074**	0.044	0.012	0.073**	0.044	0.012
	(2.019)	(1.325)	(1.476)	(2.016)	(1.320)	(1.460)
*_cons*	8.852***	11.041***	11.954***	8.865***	11.055***	11.933***
	(5.352)	(6.645)	(5.869)	(5.382)	(6.676)	(5.872)
*Firm*	*Yes*	*Yes*	*Yes*	*Yes*	*Yes*	*Yes*
*Year*	*Yes*	*Yes*	*Yes*	*Yes*	*Yes*	*Yes*
*Industry × Year*	*Yes*	*Yes*	*Yes*	*Yes*	*Yes*	*Yes*
*n*	3,408	3,408	2,908	3,408	3,408	2,908
*Adj. R^2^*	0.348	0.357	0.339	0.348	0.357	0.339

*, **, and *** indicate significance at the 10, 5, and 1% levels, respectively.

## Conclusions

Using data from A-share companies listed in the SSE and SZSE from 2007 to 2018, this study explored the relationship between chairpersons’ Down to the Countryside experiences and stock price crash risk. The results demonstrate that chairpersons with Down to the Countryside experiences (vs. those without) are more likely to adopt stable business strategies, significantly reducing stock price crash risk. The effect of chairpersons’ Down to the Countryside experiences is not significant in SOEs, likely owing to SOEs’ management selection and supervision process, which may have made chairpersons more vigilant. Moreover, the effect of Down to the Countryside experiences is more prominent among chairpersons not approaching retirement and re-appointed chairpersons as well as those in companies with sound governance practices. This study corrected for the self-selection bias and the findings regarding the effect were supported. Furthermore, changes in chairpersons were introduced as an exogenous event to conduct a DID test. The results reveal that replacing non-*Zhiqing* chairpersons with *Zhiqing* chairpersons significantly reduces stock price crash risk. Robustness and placebo tests confirm that the original conclusions are reliable and robust. Moreover, this study empirically demonstrates that risk-taking ability is a potential mediating variable between chairpersons’ experiences and stock price crash risk. Additionally, these experiences promote firm value by reducing stock price crash risk; however, the effect of the reduced stock price crash risk tends to decline over time and disappears after the second year.

This study supplements existing research on the impact of executives’ early life experiences on corporate decision making, and uncovers the positive effects of Down to the Countryside experiences. The influence on chairpersons is conducive to exerting the positive effects of a hard-work ethic, formed during the implementation of the Down to the Countryside movement, thereby creating a corporate culture of perseverance, courage, humbleness, and prudence. With the decline in the number of *Zhiqing* chairpersons, it is necessary to learn from and inherit their hard-working, meticulous, and cautious mentalities. Furthermore, it is important to be alert to the likely risk of stock price crashes during the replacement period of chairpersons to ensure that companies can continue to develop steadily and effectively while inheriting the high-quality entrepreneurial attitudes of their predecessors.

## Data availability statement

The data supporting reported results are in Chinese and are available upon request from the corresponding author.

## Author contributions

MW and YZ contributed to the conception, design of the study, performed the statistical analysis, and wrote the first draft of the manuscript. MW and DS revised the manuscript. All authors approved the submitted version.

## Footnotes

1 In Chinese history, Red Guards are groups of militant university and high school students formed into paramilitary units as part of the Cultural Revolution (1966–76). These young people often wore green jackets similar to the uniforms of the Chinese army at the time, with red armbands attached to one of the sleeves. While engaging in marches, meetings, and frenzied propagandizing, Red Guard units attacked and persecuted local party leaders as well as schoolteachers and school officials, other intellectuals, and persons of traditional views.

2 “Barefoot doctor” is a term that began to appear in the middle of the “Cultural Revolution” in the 1960s and 1970s. It refers to medical staff with certain medical knowledge and ability who were generally approved and assigned by rural or grass-roots government and were directly led and guided by the local township health centers. Their skills consisted of a mix between agriculture and medicine; they practiced agriculture during busy times and medicine in idle times; others practiced agriculture in the daytime and provided rural inhabitants with grass-roots part-time medical attention at night.

3 Workpoint is the unit that has been used to calculate the workload and labor remuneration of members of agricultural production cooperatives and people’s communes throughout Chinese history. The workpoint is the average value of the total output value of the labor unit divided by the number of people, and then shared equally to the registered work price obtained on working days except holidays.

4 Baidu Baike is an online encyclopedia owned by Baidu, a Chinese search engine.

5 In the former household registration system, a binary structure was in effect for a long time. It consisted of the city-registered residences (urban household registration or non-agricultural household registration) and rural households (agricultural household registration). Rural household residents could own their own homestead and agricultural land.
